# Trajectory, interactions, and predictors of higher symptom burden during induction therapy for multiple myeloma

**DOI:** 10.1186/s41687-024-00817-6

**Published:** 2024-12-04

**Authors:** Mona Kamal, Qiuling Shi, Shu-En Shen, Charles Cleeland, Xin Shelley Wang

**Affiliations:** 1 Department of Symptom Research, Unit 1450, The University of Texas MD Anderson Cancer Center, 1515 Holcombe Boulevard, Houston, TX 77030 USA; 2 Chongqing Medical University, Chongqing, China

**Keywords:** Symptom burden, Multiple myeloma, Induction therapy, Patient-reported outcomes (PROs)

## Abstract

**Background:**

Patients with multiple myeloma (MM) experience disabling symptoms that are difficult to manage and may persist after induction therapy. Monitoring disease-related and induction therapy–induced symptoms and identifying patients at greater risk for high symptom burden are unmet clinical needs. The objective of this study was to examine the trajectories of symptom severity over time and identify predictors of high symptom burden during MM induction therapy.

**Methodology:**

Eligible patients with MM rated their symptoms by completing the MD Anderson Symptom Inventory MM module repeatedly during 16 weeks of induction therapy. Group-based trajectory modeling identified patient groups with persistently high-severity (versus low-severity) symptom trajectories over time. Quality of life (QOL) and affective and physical functioning status were assessed. Predictors of high symptom burden were examined by regression analysis.

**Results:**

Sixty-four MM patients participated. Most patients (89%) received bortezomib-based therapy. The five most-severe symptom trajectory groups were pain (59%), muscle weakness (46%), numbness (42%), disturbed sleep (41%), and fatigue (31%). Patients in the high-severity trajectory group for the five most-severe symptoms (31% of the sample) were more likely to have high-severity cognitive and affective symptoms. Patients in the high-severity trajectory groups for fatigue, muscle weakness, disturbed sleep, and bone aches were more likely to have high pain scores (all *p* < 0.05). Significant increases over time were observed in scores for pain (estimate: 0.026), numbness (0.051), muscle weakness (0.020), physical items (0.028), and affective items (0.014) (all *p* < 0.05). A higher baseline composite score of the five most-severe symptoms predicted worse QOL (− 6.24), and poor affective (0.80) and physical (1.10) statuses (all *p* < 0.01). Female sex predicted higher risk for being in the high-severity trajectory group for muscle weakness.

**Conclusion:**

Almost one-third of MM patients suffer from up to 5 moderate to severe symptoms persistently, including pain, muscle weakness, numbness, disturbed sleep, and fatigue. Importantly, these results identify a group of symptoms that should be monitored and managed as part of routine patient care during MM induction therapy and suggest that pre-therapy pain management is necessary for better symptom control.

**Supplementary Information:**

The online version contains supplementary material available at 10.1186/s41687-024-00817-6.

## Introduction

Multiple myeloma (MM) accounts for approximately 17% of all hematological cancers [1, 2]. In 2023, an estimated 35,730 new cases of MM will be identified in the United States [3]. Bortezomib-based induction therapy has improved tumor response substantially [4] and remains the standard-of-care option for patients with MM [5]. However, disabling symptoms related to MM [6] or its treatment [7] are difficult to manage and may persist during and after the treatment course [8]. Moreover, many patients with MM undergo hematopoietic stem-cell transplant (HSCT) immediately after induction therapy, while they are still experiencing induction therapy–related symptoms. For these reasons, consideration should be given to incorporating patient-reported outcome (PRO)-based symptom assessment and management [9] into routine care for MM patients before HSCT [10].

Among MM patients receiving standard induction therapy, symptom burden can vary depending on the patient’s tumor burden, general condition, comorbidities, and treatment- induced toxicities [11] and can negatively affect patients’ physical status and quality of life (QOL) [6]. Consequently, there is a growing research effort to highlight the need for including PROs as outcomes in clinical trials [12] and for capturing symptom severity from the patient’s perspective, to ensure better outcomes from personalized MM treatment plans [13].

Increased attention is now being given to PROs as a means of accounting for patients’ subjective experiences of symptom severity, QOL, and functional status during MM treatment [14]. Patient perspectives can now be assessed by using validated, standardized, disease-specific questionnaires. Such tools are valuable for judging the impact of disease burden and treatment on a patient’s well-being [15]. The use of subjective PRO measures avoids observer bias and, therefore, can help clinicians make better treatment decisions for patients with MM [14]. In addition, the US Food and Drug Administration has endorsed the use of PRO measures in clinical trials to support labeling claims for approved medical products [12].

One such validated multiple-symptom PRO assessment tool is the MD Anderson Symptom Inventory (MDASI) [16]. Previous work using the MM module of the MDASI (MDASI-MM) [17] has shown the tool’s promising effectiveness and sensitivity in capturing the symptom burden of patients with MM who were receiving standard-of-care HSCT or maintenance therapy [18–20]. Nonetheless, no detailed, dedicated study has evaluated symptom burden during induction therapy for MM.

The present prospective longitudinal study used the MDASI-MM to characterize the trends in symptom development and its impact from the patient’s perspective during MM induction therapy and to evaluate the interaction of multiple MM symptoms, as a reflection of the effects of tumor burden and treatment received. The current study also investigated the potential value of using baseline symptom burden to predict the risk for developing high symptom burden over time. Such data may support proactive risk-based treatment strategies that improve treatment tolerance among patients with MM receiving induction therapy and, potentially, improve long-term oncologic outcomes in patients with MM.

## Materials and methods

This prospective, longitudinal study was approved by the Institutional Review Board of The University of Texas MD Anderson Cancer Center.

### Patients

Patients with newly diagnosed MM at MD Anderson Cancer Center who were scheduled to receive bortezomib-based induction chemotherapy as standard care with or without lenalidomide were screened for eligibility. However, patients who had received steroid therapy only or had undergone no more than two previous cycles of induction therapy for MM also were eligible. Patients with preexisting peripheral neuropathy were excluded, but patients with preexisting diabetes were not.

At enrollment, the following patient characteristics were collected: age, sex, race, education level, disease stage, comorbidities, Eastern Cooperative Oncology Group performance status (ECOG-PS) [21, 22], body mass index, opioid use, pre-induction therapy treatment status, pre-induction therapy tumor response, baseline hemoglobin level, diabetes status, and induction chemotherapy regimen. The ECOG-PS score reflects patient functioning in terms of self-care, daily activity, and physical ability (walking, working, etc.); a score of 0–1 indicates good performance status, and a score of 2–5 indicates poorer performance status. Comorbidities were summarized using the Charlson Comorbidity Index [23, 24], which considers 19 pre-defined comorbid conditions to predict mortality risk and provides a weighted score based on the number and severity of these comorbidities. The opioid information was available from baseline to 16 weeks after induction therapy initiation.

### Symptom assessment measures

The psychometrically validated MDASI-MM [17, 18] was used to assess patients’ subjective symptom burden resulting from multiple general cancer symptoms and MM-specific symptoms. Patients rated their symptoms at their worst over the past 24 h from 0 (“not at all present”) to 10 (“as bad as you can imagine”) during induction therapy twice a week for 12 weeks and then weekly for up to 16 weeks. Cognitive symptoms include difficulty with paying attention and remembering; affective symptoms include distress and sadness.

The six interference items of the MDASI-MM were used to measure symptom interference with functioning. Three interference items (work, activity, and walking) measure physical status, and three items (relations with others, enjoyment of life, and mood) measure affective status. Consistent with the symptom ratings, all six symptom interference items are rated with a recall of the last 24 h, on a 0–10 scale.

The European Organization for Research and Treatment of Cancer (EORTC)-QLQ-C30 questionnaire [25] was used to assess global QOL and physical, role, emotional, cognitive, and social functioning status at baseline.

### Statistical analysis

Frequencies were assessed for all categorical covariates. Means, standard deviations, medians, and ranges were calculated for all continuous covariates.

Trends in mean score of symptom reduction or worsening over time were examined using mixed-effects modeling. The five most-severe symptoms were identified by their having the highest mean scores of reported MDASI-MM symptom items across all time points. Composite scores [26] were calculated by summing the mean scores of all symptom items of interest over time and dividing that sum by the number of items. Composite scores were calculated in this way for the five most-severe symptoms, the two MDASI-MM cognitive symptoms, and the two MDASI-MM affective symptoms. Similarly, composite scores were calculated for the MDASI-MM’s six interference items and each of the three-item interference subscales (physical items and affective items).

Group-based trajectory modeling (GBTM) estimates symptom patterns over time and identifies subgroups of patients with similar symptom reporting trajectories [27]. For this study, GBTM was used to identify groups of patients who persistently reported high symptom severity over time (moderate-to-severe symptom scores on the MDASI-MM) (high-severity trajectory groups) [28] and groups who consistently reported low symptom severity (mild symptom scores on the MDASI-MM) over time (low-severity trajectory groups) [29]. The percentages of patients in these two types of symptom trajectory group are reported.

In addition, GBTM results were used to determine baseline predictors for symptom severity development and to study the interactions among high-severity symptom trajectory groups. The EORTC-QLQ-C30 score was used at baseline to predict the risk for being in the high-severity trajectory group.

Multivariate logistic regression was used to adjust the model for the following patient and clinical factors: age (with ≥ 70 years as the cutpoint), sex, diabetes, anemia, body mass index, comorbidities, disease stage, ECOG-PS, previous treatment (steroids only, one or two cycles of chemotherapy, or treatment naïve), tumor response, opioid use, and chemotherapeutic regimens.

All statistical analyses were conducted using SAS 9.3 (SAS Institute, Inc., Cary, NC).

## Results

### Participants

Between May 2008 and March 2011, 64 patients were enrolled in the study. The sample was primarily male (59%) and non-Hispanic White (73%). The mean age was 63 years; 47% of patients were at least 65 years of age, and 17% were at least 75 years of age. Most (89%) of the patients received bortezomib-based induction therapy. Twenty-one patients had completed the MDASI-MM at week sixteen, yielding a dropout rate of 67.2% at the end of the study. See Table 1 for patient demographic and clinical characteristics.

**Table 1:** Demographic and clinical characteristics of patients with multiple myeloma undergoing induction therapy

Characteristic	*n*	Mean (SD)	Median (Range)
Age, years	64	63 (12.0)	63 (24–86)
Charlson comorbidity index	64	0.84 (1.41)	0 (0–8)
Body mass index, kg/m^2^	55	28 (10.3)	28 (24–57)
**Characteristic**	***n***	**%**	
Age, years (*n* = 64)			
< 65	34	53	
≥ 65	30	47	
≥ 75	11	17	
Sex (*n* = 64)			
Male	38	59	
Female	26	41	
Race (*n* = 64)			
Non-Hispanic white	47	73	
Other	17	27	
Highest education level (*n* = 64)			
College or higher	50	78	
Middle or high school	14	22	
Disease stage (*n* = 64)			
I	27	42	
II	20	31	
III	17	27	
ECOG-PS (*n* = 55)			
0	12	22	
1	34	62	
2	8	14	
3	1	2	
Body mass index (*n* = 55)			
< 30	36	65	
≥ 30 (obese)	19	35	
Opioid use (*n* = 64)			
Yes	33	52	
No	31	48	
Previous therapy (*n* = 64)			
Treatment-naïve	23	36	
Steroids only	4	6	
1 chemotherapy cycle	27	42	
2 chemotherapy cycles	10	16	
Tumor response at the end of induction therapy (*n* = 62)			
Complete response	4	6	
Stringent complete response	1	2	
Very good partial response	30	48	
Partial response	16	26	
Stable disease	10	16	
Relapse after complete response	1	2	
Baseline hemoglobin, g/dL (*n* = 62)			
≤ 11	30	48	
> 11	32	52	
Diabetes diagnosis (*n* = 64)			
No	54	84	
Yes	10	16	
Induction therapy regimen (*n* = 64)			
Bortezomib only	2	3	
Dexamethasone only	7	11	
Bortezomib and dexamethasone	49	77	
Bortezomib, dexamethasone, and thalidomide	6	9	

*ECOG-PS*, Eastern Cooperative Oncology Group performance status; *SD*, standard deviation

### Symptom severity at baseline and over time

#### Baseline symptom burden

The MDASI-MM symptoms with the highest mean (SD) severity scores at baseline were fatigue, 4.34 (2.70); pain, 3.48 (3.14); drowsiness, 3.13 (2.86); disturbed sleep, 3.08 (2.94); bone aches, 2.97 (3.02); dry mouth, 2.66 (3.16); and muscle weakness, 2.28 (2.79). See Supplementary Table 1.

#### Symptom development over time, by univariate and multivariate analysis

**Fig. 1:**
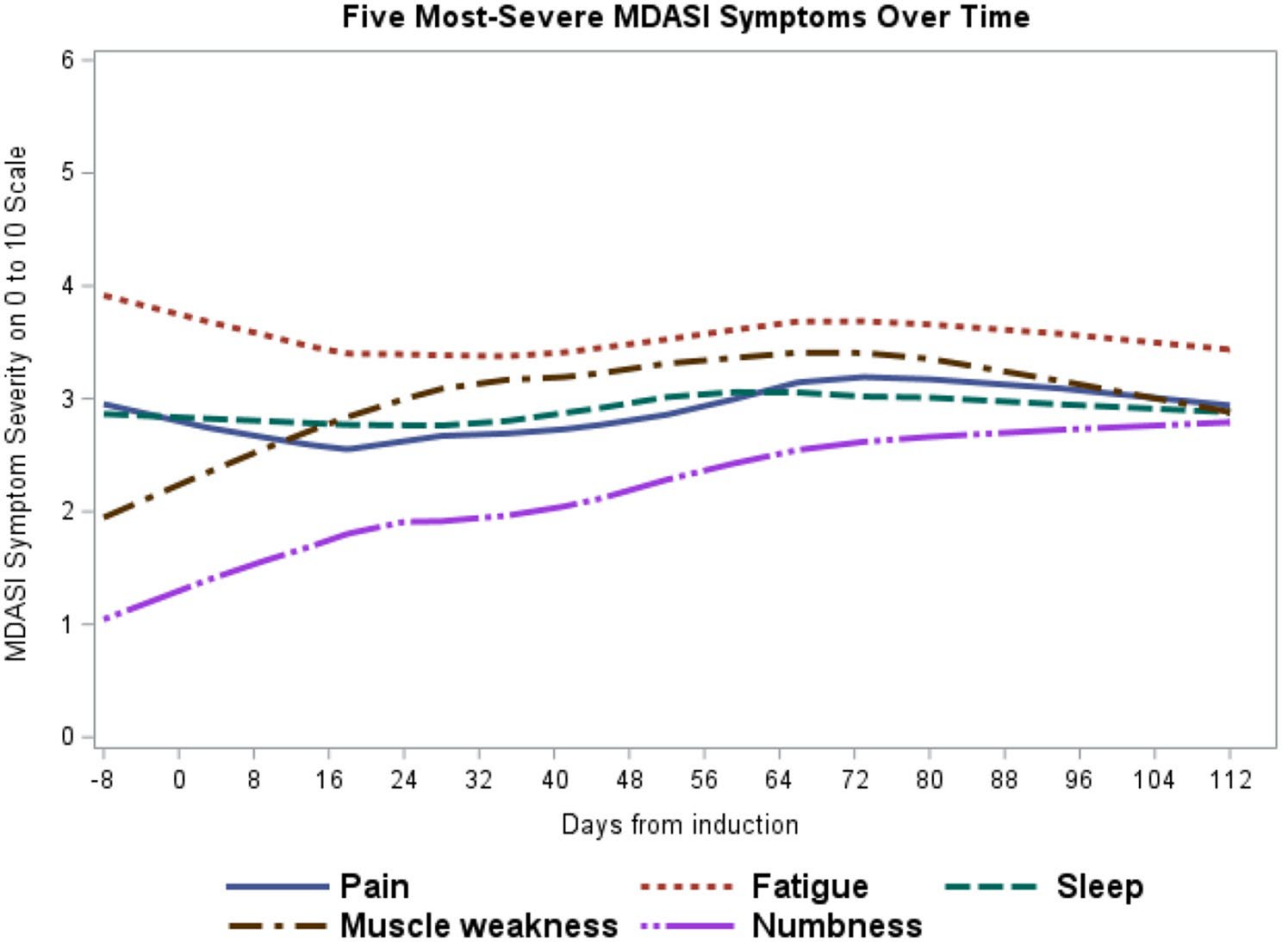
Symptom development patterns over time during induction therapy for patients with multiple myeloma undergoing induction therapy (*n* = 64). Loess curves depicting the mean severity over time of the five most severe symptoms during induction therapy, as reported using the MD Anderson Symptom Inventory multiple myeloma module (MDASI-MM). Results from mixed modeling analysis indicated that the severity of pain, numbness, and muscle weakness increased significantly over time, whereas the severity of fatigue and sleep disturbance did not change significantly over time

After induction therapy was initiated, fatigue, pain, muscle weakness, numbness, and disturbed sleep were the five most severe symptoms over time (Fig. 1). A longitudinal univariate model revealed the dynamic changes in symptom severity over time. Severity scores increased for pain (estimate [Est] = 0.017; *p =* 0.01), numbness (Est = 0.044; *p* < 0.001), and muscle weakness (Est = 0.021; *p =* 0.001), decreased for drowsiness (Est = − 0.016; *p =* 0.02), and did not change for fatigue (Est = 0.0002; *p =* 0.98), sleep disturbance (Est = − 0.0003; *p =* 0.96), and bone aches (Est = 0.0004; *p =* 0.95) over time. See Supplementary Table 2.

For all patients, multivariate mixed modeling controlled for age (< 70 vs. ≥ 70 years), sex, disease stage, ECOG-PS, previous treatment, tumor response, and opioid use found significant increases from baseline over time in the severity of pain (Est = 0.026; *p =* 0.007), numbness (Est = 0.051; *p =* 0.007), muscle weakness (Est = 0.020; *p =* 0.007), and rash (Est = 0.012; *p =* 0.004).

In mixed-effects modeling, an increase in numbness during induction therapy was significantly associated with concomitant increases in the severity of pain (*p* = 0.005), disturbed sleep (*p* < 0.001), and muscle weakness (*p* < 0.001).

### Trajectories and interactions among the five most-severe symptoms over time

**Fig. 2:**
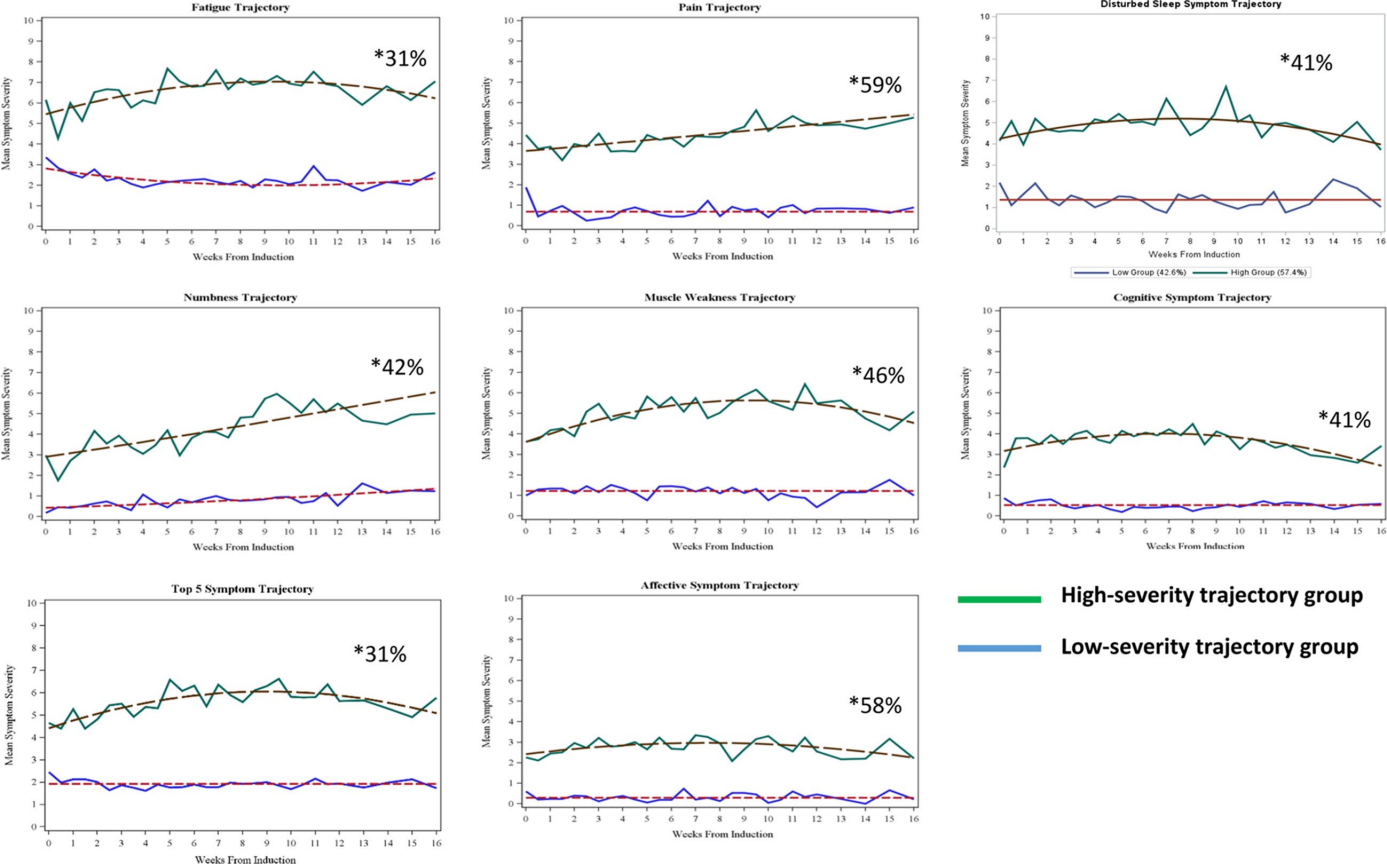
High-severity vs. low-severity symptom trajectory groups among patients with multiple myeloma undergoing induction therapy (*n* = 64). Group-based trajectory modeling of the mean severity of MDASI-MM items over time, grouped by percentage of patients who self-reported symptom and interference items as more-severe versus those who reported those items as less-severe. Cognitive symptoms include difficulty paying attention and difficulty remembering. Affective symptoms include distress and sadness. The five most-severe symptoms over time were fatigue, pain, muscle weakness, numbness, and disturbed sleep. * The percentage of patients in the high-severity symptom trajectory group. For example, for fatigue, 31% of the patients persistently reported higher fatigue scores over time, compared with the remaining 69% of patients

Based on GBTM, Fig. 2 presents the percentages of patients who persistently reported high versus low symptom burden for the five most-severe MDASI-MM symptoms (fatigue, pain, numbness, muscle weakness, and disturbed sleep) over time, both individually and collectively, and for self-reported cognitive and affective symptoms.

In the GBTM analysis, some patients were persistently in a high-severity trajectory group: 31% of the patients for fatigue, 59% of patients for pain, 46% of patients for muscle weakness, 42% of patients for numbness, 41% of patients for disturbed sleep, 41% of patients for cognitive symptoms, and 58% of patients for affective symptoms; 31% were in the high-severity trajectory group for the five most-severe symptoms (Fig. 2).

Patients in the high-severity trajectory group for the five most-severe symptoms were more likely than those in the low-severity trajectory group to have high symptom burdens for cognitive symptoms and affective symptoms. Patients in the high-severity trajectory group for fatigue, muscle weakness, disturbed sleep, or bone aches were more likely to have high pain scores (all *p* < 0.05; Table 2).

**Table 2:** Interactions among high-severity symptom trajectory groups* during induction therapy

Prevalence of high symptom burden (% of patients with persistently higher PRO scores)*	Fatigue	Pain	Muscle weakness	Numbness	Cognitive symptoms	Affective symptoms	Five-item composite score
Five most-severe symptoms** (31%)	95	85		65	75	90	
Fatigue (31%)**		85			75	90	
Pain (59%)	45		60	55	50	70	
Numbness (42%)							48
Muscle weakness (45%)	65	79			65	86	69
Disturbed sleep (41%)	65	77	77	62	69	85	65
Bone aches (32%)	71	95		85	62	90	76
Cognitive symptoms (41%)***	58		69			85	58
Affective symptoms (56%)***	50	80	69		61		50

*PRO*, patient-reported outcome

* Trajectory group membership indicates the percentage of patients with similar symptom trajectories over time

** The five most-severe symptoms over time were fatigue, pain, muscle weakness, numbness, and disturbed sleep

*** Cognitive symptoms include difficulty paying attention and difficulty remembering. Affective symptoms include distress and sadness

For example, for patients with fatigue, 31% persistently reported higher fatigue scores over time, compared with the remaining 69% of those patients, and 85% of those patients reported persistently higher pain scores over time

### Patient and clinical characteristics associated with high symptom burden

After adjustment for clinical variables, the multivariate logistic regression model showed that moderate-to-severe baseline pain predicted a higher risk for being in the high-severity trajectory group for the five most-severe symptoms (*p* = 0.008) (Table 3). A high baseline drowsiness score predicted a higher risk for being in the high-severity trajectory group for fatigue (*p* = 0.007). Higher risk for being in the high-severity trajectory group for muscle weakness was associated with a high baseline pain score (*p* = 0.008) and female sex (*p* = 0.03). High baseline scores for distress, numbness, and constipation predicted a higher risk for being in the high-severity trajectory group for disturbed sleep (*p* < 0.05). A high baseline fatigue score was associated with a higher risk for being in the high-severity trajectory group for bone aches (*p* = 0.03).

**Table 3:** Predictors of high-symptom trajectory group membership*

Baseline predictor	Outcomes	Odds ratio (95% CI)	*p* value
Pain score	High-severity group membership for the five most-severe symptoms**	6.250 (1.63 − 24.02)	0.008
Drowsiness score	High-severity trajectory group membership for fatigue	6.214 (1.66 − 23.31)	0.007
Dry mouth score	High-severity trajectory group membership for pain	6.023 (1.37 − 26.54)	0.02
Female sex	High-severity trajectory group membership for numbness	0.214 (0.05 − 0.91)	0.04
Fatigue score	High-severity trajectory group membership for bone aches	4.666 (1.13 − 19.24)	0.03

*MDASI-MM*, MD Anderson Symptom Inventory multiple myeloma module

* Trajectory group membership indicates the percentage of patients with similar symptom trajectories over time

** The five most-severe MDASI-MM items over time were fatigue, pain, muscle weakness, numbness, and disturbed sleep

Female sex (odds ratio [OR] 15.103, 95% CI 2.46–92.56; *p* = 0.003) and opioid use (OR 6.941, 95% CI 1.21–39.82); *p* = 0.03) predicted higher risk for being in the high-severity trajectory group for affective symptoms. However, such wide 95% CI results may indicate poor stability of the statistical model and affect the generalizability of the regression prediction model results.

### Symptom burden impact on functioning and relation to QOL

Overall, patient functional status worsened over the course of induction therapy, as evidenced by the significant increases in the composite scores of total interference on MDASI-MM (Est = 0.021; *p =* 0.006), physical status (work, activity, and walking) (Est = 0.028; *p =* 0.007), and affective status (relations with others, enjoyment of life, and mood) (Est = 0.014; *p =* 0.006) in univariate modeling. Over time, univariate analysis showed that a higher baseline composite score for the five most-severe symptoms predicted worse affective status (Est = 0.80; *p* < 0.001) and worse physical status (Est = 1.10; *p* < 0.001).

Approximately 37% and 44% of the patients reported moderate to severe EORTC-QLQ-C30 scores for global QOL and emotional function at baseline, respectively. A higher baseline mean composite score for the five most-severe symptoms at baseline was associated with worse QOL status (measured using the global QOL score from the EORTC-QLQ-C30, − 6.24; *p* = 0.01).

A poor EORTC-QLQ-C30 score (i.e., lower than the median score) for emotional function at baseline predicted an increased risk for being in the high-severity trajectory group for the five most-severe MDASI-MM symptoms and the affective symptoms. Also, a poor EORTC-QLQ-C30 global QOL score at baseline predicted increased risk for being in the high-severity trajectory group for the five most-severe MDASI-MM items. A poor EORTC-QLQ-C30 cognitive function score at baseline predicted an increased risk for being in the high-severity trajectory group for cognitive symptoms and for affective symptoms. A poor EORTC-QLQ-C30 social function score at baseline predicted an increased risk for being in the high-severity trajectory group for affective symptoms. See Table 4.

**Table 4:** Baseline dichotomized EORTC-QLQ-C30 scores* predict membership in high-severity symptom trajectory groups during induction therapy for multiple myeloma

EORTC-QLQ-C30 domain	MDASI-MM high-severity trajectory group						
	5 most-severe OR (95% CI)	Cognitive OR (95% CI)	Affective OR (95% CI)	Pain OR (95% CI)	Fatigue OR (95% CI)	Numbness OR (95% CI)	Muscle weakness OR (95% CI)
Global QOL	**3.55** **(1.15–10.98)**	2.18 (0.76–6.27)	2.67 (0.90–7.92)	**3.42** **(1.06–11.05)**	**3.55** **(1.15–10.98)**	0.83 (0.29–2.38)	2.78 (0.96–8.04)
Physical function	2.29 (0.78–6.78)	2.24 (0.81–6.23)	2.14 (0.77–5.93)	**4.86** **(1.58–14.96)**	1.70 (0.58–4.95)	1.16 (0.43–3.16)	2.60 (0.94–7.20)
Role function	1.38 (0.47–4.05)	1.58 (0.57–4.41)	1.78 (0.63–5.01)	1.72 (0.60–4.95)	1.38 (0.47–4.05)	0.63 (0.22–1.76)	1.95 (0.70–5.43)
Emotional function	**6.92** **(2.08–23.06)**	2.52 (0.90–7.05)	**13.09** **(3.65–46.92)**	**4.35** **(1.42–13.36)**	**6.92** **(2.08–23.06)**	1.00 (0.36–2.73)	**7.22** **(2.36–22.06)**
Cognitive function	1.94 (0.63–5.99)	**5.17** **(1.61–16.55)**	**7.02** **(1.78–27.60)**	**5.33** **(1.36–20.93)**	2.70 (0.87–8.36)	1.77 (0.60–5.23)	1.99 (0.67–5.91)
Social function	3.09 (0.98–9.73)	1.65 (0.55–4.96)	**10.95** **(2.25–53.39)**	3.06 (0.87–10.75)	3.09 (0.98–9.73)	0.80 (0.26–2.42)	2.36 (0.77–7.22)

*EORTC*, European Organisation for Research and Treatment of Cancer; *MDASI-MM*, MD Anderson Symptom Inventory for multiple myeloma; *OR*, odds ratio; *QOL*, quality of life

* Dichotomized EORTC score (good: median or higher; poor: lower than median)

Boldface indicates a statistically significant value

## Discussion

In this study of patient-reported MM disease-related and treatment-induced symptom burden in patients who predominantly received bortezomib-based induction therapy, fatigue was persistently the most severe symptom during therapy; the other symptoms with the greatest severity during therapy were muscle weakness, disturbed sleep, pain, drowsiness, bone aches, and numbness. Fatigue and neuropathy are significant concerns for patients with MM and for cancer providers because of their negative impact on QOL [30]. Moreover, bone aches, muscle weakness, pain, numbness, and fatigue due to disease activity (lytic lesions, compressed discs, renal insufficiency, anemia) are typical clinical manifestations of MM that negatively impact physical status and QOL [31, 32].

In our study cohort, the median age was 63 years, 17% of patients were at least 75 years old, and 16% had an ECOG-PS of 2 or 3 at baseline. There are more elderly MM survivors today than in the past, and elderly survivors are more susceptible to treatment-induced and disease-related toxic effects and comorbid conditions than are younger survivors. Given that most patients with MM are elderly [2] and have a compromised ECOG-PS due to concurrent comorbidities [33], these vulnerable patients need personalized treatment plans and close monitoring [34].

This study used trajectory analysis to provide a detailed description of the similarity of the most important and persistently high-severity symptom burden over time in patients receiving induction therapy for MM. An advantage of trajectory analysis for examining longitudinal PROs is that it can track the severity and prevalence of a targeted symptom over time, thereby providing useful information about the percentage of patients who need close monitoring and care [29].

Neuropathy is a debilitating presenting symptom of MM that can worsen with treatment. In this study, 42% of the patients with MM reported a rapid increase in the severity of numbness, a neuropathy symptom that was significantly associated with pain, disturbed sleep, and muscle weakness. Because preexisting neuropathy was one of our exclusion criteria, our results reflect the burden of chemotherapy-induced neuropathy from bortezomib-based induction therapy, which 89% of patients received [35]. Moreover, inherent exaggerating factors (disease activity, advanced age, comorbid conditions) can exacerbate peripheral neuropathy in patients with MM [36–38]. Treatment-induced peripheral polyneuropathy may present as motor symptoms or as sensory or autonomic deficits (in the form of muscle weakness, painful neuropathy, or orthostatic hypotension) and may worsen the existing muscle weakness, pain, and drowsiness, which may explain why patients in our high-severity trajectory group for numbness had high composite MDASI-MM scores [39, 40]. New MM treatment agents induce significant dose-dependent neuropathy with prolonged recovery times; with the introduction of these new treatment agents, iatrogenic neurotoxicity has become the leading cause of peripheral neuropathy [30, 35]. Nonetheless, the incidence and severity of iatrogenic neurotoxicity in patients with MM have decreased owing to dose-reduction guidelines and increased awareness of treatment-induced peripheral neuropathy [41, 42].

Patients with MM often have to take opioids, not only resolve treatment-induced peripheral neuropathy [43, 44], but also to relieve bone aches [45]. In this study, opioid use predicted an increased risk for being in the high-severity trajectory group for affective symptoms (although the 95% CIs were wide, which may have impacted the generalizability of the regression prediction model results), and patients with high baseline pain scores were at increased risk for being in the high-severity trajectory group for the five most-severe symptoms. Opioid use compromises QOL in patients with MM [46] and carries risks for constipation, breakthrough pain requiring hospitalization, and opioid misuse and tolerance [47, 48]. That said, others have argued that opioids may contribute to the QOL improvement in patients with MM by relieving severe pain, as well as by relieving the mood changes associated with such severe pain [49]. Optimized use of opioids in patients with MM is especially necessary in those with renal impairment or other organ dysfunctions [50, 51]. These differing opinions regarding the use and benefits of opioids in MM patients support the optimization of opioid use and close monitoring and managing of its side effects.

Clinically, patients rarely suffer from a single severe symptom. Interestingly, multiple interactions among high-severity symptom burden groups were observed in the current study, such as interactions between patients with high pain scores and those with high scores for muscle weakness, bone aches, numbness, fatigue, and cognitive and affective symptoms. Patients in the high-severity trajectory groups for muscle weakness and bone aches also reported more severe pain, fatigue, cognitive symptoms, and affective symptoms and had higher composite scores for the five most-severe MDASI-MM items. Patients in the high-severity trajectory group for fatigue also had more severe pain and bone aches and worse cognitive and affective status. High baseline drowsiness scores were associated with an increased risk for being in the high-severity group for fatigue over time. These findings emphasize the potential intercurrent or concurrent relationship between the burdens of pain, fatigue, and cognitive and functional impairment in patients with MM [52–55]. Of note, disrupted sleep was found to be one of the most frequently reported MDASI-MM items, suggesting that sleep disturbance is an independent symptom that should be monitored and triaged for clinical action. Indeed, it could be an impact of the disease/treatment too; for example, it is an item on the Brief Pain Inventory [56].

This study provides a comprehensive description of the symptom trajectories and predictors of symptom burden during induction therapy for patients with MM. Our study presents a novel approach for interpreting symptom clusters driven by both disease and the standard-of-care treatment regimen and was strengthened by its longitudinal study design and analysis methods. However, the general applicability of the findings may be limited due to the well-educated population and lack of ethnic/racial diversity in this single-institution study. Also, the impacts of compliance with pain medications, changing medications, and interruption of pain medications are not easily tracked by using PRO measures, and these compliance issues could have affected our findings.

The overlapping effects of patient, disease, and treatment characteristics on PROs need to be considered when interpreting similar data in future studies. Our data showed the statistical interpretation of our findings, which will be shared with the clinicians through this publication for their clinical interpretation and insights. Further analysis, such as calculating the minimally important differences for individual symptoms and composite scores and the critical PRO cut points linked to clinical outcomes, would guide data interpretation in the clinical setting. However, such analyses are out of the scope of this report. How to interpret the small mean changes clinically at a group level remains unclear. Further, the relatively high dropout rate of 67.2% at the end of the study mirrored real-world findings. Electronic PRO data collection methods could improve this in future symptom studies.

## Conclusion

Patients with MM undergoing induction therapy suffer from a cluster of persistently severe symptoms that interfere with daily functioning and negatively impact QOL. Incorporating these well identified PROs into the personalized treatment plans of patients with MM are needed to improve outcomes, increase treatment tolerability, and adapt to the challenges of symptom burden, aging, and comorbid conditions during induction therapy. Implementing such strategies might also help improve patient outcomes after HSCT [57–59]. Pre-therapy pain management may be important for better symptom control in this patient cohort.

## Electronic supplementary material

Below is the link to the electronic supplementary material.

Supplementary Material 1

## Data Availability

Not applicable.
