# Supplementary material for: Trajectory, interactions, and predictors of higher symptom burden during induction therapy for multiple myeloma
Source: J Patient Rep Outcomes. 2024 Dec 4;8:141. doi: 10.1186/s41687-024-00817-6 (PMC11618278; doi:10.1186/s41687-024-00817-6)
Supplement: Supplementary file 1 — Supplementary Material 1 [file 41687_2024_817_MOESM1_ESM.docx]

**Supplementary Table 1. Baseline descriptive statistics for the MDASI-MM symptom and interference items**

| **MDASI-MM items** | **N** | **Mean** | **SD** | **Median** | **Min** | **Max** | **% ≥4** |
| --- | --- | --- | --- | --- | --- | --- | --- |
| Pain | 64 | 3.5 | 3.1 | 3 | 0 | 10 | 43.8 |
| Fatigue | 64 | 4.3 | 2.7 | 4.5 | 0 | 10 | 62.5 |
| Nausea | 64 | 0.4 | 1.1 | 0 | 0 | 5 | 1.6 |
| Sleep disturbance | 64 | 3.1 | 2.9 | 2.5 | 0 | 10 | 42.2 |
| Distress | 64 | 1.9 | 2.4 | 1 | 0 | 10 | 20.3 |
| Shortness of breath | 64 | 1.2 | 2.1 | 0 | 0 | 8 | 12.5 |
| Difficulty remembering | 64 | 1.9 | 2.2 | 1 | 0 | 10 | 15.6 |
| Lack of appetite | 63 | 1.0 | 1.8 | 0 | 0 | 7 | 14.3 |
| Drowsiness | 64 | 3.1 | 2.9 | 3 | 0 | 10 | 32.8 |
| Dry mouth | 64 | 2.7 | 3.2 | 2 | 0 | 10 | 32.8 |
| Sadness | 64 | 1.3 | 2.2 | 0 | 0 | 8 | 17.2 |
| Vomiting | 64 | 0.0 | 0.0 | 0 | 0 | 0 | 0.0 |
| Numbness | 64 | 1.4 | 2.4 | 0 | 0 | 9 | 14.1 |
| Constipation | 64 | 2.0 | 2.9 | 0 | 0 | 9 | 21.9 |
| Muscle weakness | 64 | 2.3 | 2.8 | 1 | 0 | 10 | 31.3 |
| Diarrhea | 64 | 0.2 | 0.8 | 0 | 0 | 4 | 1.6 |
| Mouth/throat sores | 64 | 0.9 | 1.6 | 0 | 0 | 6 | 9.4 |
| Rash | 62 | 0.3 | 1.4 | 0 | 0 | 8 | 3.2 |
| Difficulty paying attention | 64 | 1.0 | 1.6 | 0 | 0 | 6 | 9.4 |
| Bone aches | 64 | 3.0 | 3.0 | 3 | 0 | 10 | 40.6 |
| All interference items | 64 | 2.6 | 2.4 | 1.75 | 0 | 8 | 31.3 |
| WAW* | 64 | 3.2 | 2.9 | 2.33 | 0 | 9 | 39.1 |
| REM** | 64 | 2.0 | 2.2 | 1.33 | 0 | 7 | 20.3 |
| Cognitive symptoms*** | 64 | 1.5 | 1.7 | 1 | 0 | 8 | 10.9 |
| Affective symptoms**** | 64 | 1.6 | 2.1 | 1 | 0 | 9 | 12.5 |

*MDASI-MM*, MD Anderson Symptom Inventory for multiple myeloma.

* WAW includes the interference items work, general activity, and walking, as a representation and measure of physical status.

** REM includes the interference items relations with others, enjoyment of life, and mood, as a representation and measure of affective status.

*** Cognitive symptoms include difficulty paying attention and difficulty remembering.

**** Affective symptoms include distress and sadness.

**Supplementary Table 2. Longitudinal mixed modeling of symptom and interference items from the MDASI-MM**

| **MDASI-MM item** | **Estimate** | ***p* value** |
| --- | --- | --- |
| Pain | 0.0172 | **0.01** |
| Fatigue | 0.0002 | 0.98 |
| Nausea | −0.0056 | 0.14 |
| Sleep disturbance | −0.0003 | 0.96 |
| Distress | −0.0080 | 0.18 |
| Shortness of breath | 0.0041 | 0.44 |
| Difficulty remembering | −0.0066 | 0.13 |
| Lack of appetite | 0.0036 | 0.50 |
| Drowsiness | −0.0162 | **0.02** |
| Dry mouth | −0.0281 | **< 0.001** |
| Sadness | −0.0021 | 0.70 |
| Vomiting | −0.0032 | 0.18 |
| Numbness | 0.0437 | **< 0.001** |
| Constipation | −0.0141 | **0.04** |
| Muscle weakness | 0.0206 | **0.001** |
| Diarrhea | 0.0037 | 0.44 |
| Mouth/throat sores | −0.0109 | **0.02** |
| Rash | 0.0090 | **0.03** |
| Difficulty paying attention | 0.0038 | 0.42 |
| Bone aches | 0.0004 | 0.95 |
| Interference items | 0.0198 | **< 0.001** |
| WAW* | 0.0287 | **< 0.001** |
| REM** | 0.0109 | **0.04** |
| Cognitive symptoms*** | −0.0014 | 0.73 |
| Affective symptoms**** | −0.0044 | 0.29 |

*MDASI-MM*, MD Anderson Symptom Inventory for multiple myeloma.

* WAW includes the interference items work, activity, and walking, as a representation and measure of physical status.

** REM includes the interference items relations with others, enjoyment of life, and mood, as a representation and measure of affective status.

*** Cognitive symptoms include difficulty paying attention and difficulty remembering.

**** Affective symptoms include distress and sadness.
